# A New Variational Approach for Multiplicative Noise and Blur Removal

**DOI:** 10.1371/journal.pone.0161787

**Published:** 2017-01-31

**Authors:** Asmat Ullah, Wen Chen, Mushtaq Ahmad Khan, HongGuang Sun

**Affiliations:** State Key Laboratory of Hydrology-Water Resources and Hydraulic Engineering, Center for Numerical Simulation Software in Engineering and Sciences, College of Mechanics and Materials Hohai University, Nanjing, Jiangsu 210098, P. R. China; University of California Santa Barbara, UNITED STATES

## Abstract

This paper proposes a new variational model for joint multiplicative denoising and deblurring. It combines a total generalized variation filter (which has been proved to be able to reduce the blocky-effects by being aware of high-order smoothness) and shearlet transform (that effectively preserves anisotropic image features such as sharp edges, curves and so on). The new model takes the advantage of both regularizers since it is able to minimize the staircase effects while preserving sharp edges, textures and other fine image details. The existence and uniqueness of a solution to the proposed variational model is also discussed. The resulting energy functional is then solved by using alternating direction method of multipliers. Numerical experiments showing that the proposed model achieves satisfactory restoration results, both visually and quantitatively in handling the blur (motion, Gaussian, disk, and Moffat) and multiplicative noise (Gaussian, Gamma, or Rayleigh) reduction. A comparison with other recent methods in this field is provided as well. The proposed model can also be applied for restoring both single and multi-channel images contaminated with multiplicative noise, and permit cross-channel blurs when the underlying image has more than one channel. Numerical tests on color images are conducted to demonstrate the effectiveness of the proposed model.

## 1 Introduction

Image de-noising and de-blurring are two fundamental tasks in image processing and signal fields. In many real world practices, contamination effects are inevitable during image transmission and acquirement. For example, the images captured by astronomical telescopes are usually be influenced by atmospheric turbulence and results in blurred images. In order to further improve image processing tasks, image de-noising and image de-blurring continue to attract the attention of many researchers in the modern imaging sciences community [1, 2]. In the literature, various kinds of noise were considered, such as additive noise, impulse noise, Cauchy noise, and Poisson noise. The interested reader is referred to [3–15] and references therein for more details of those noise removal models and the restoration methods. Based on the imaging systems, let *u* be an original *m*_1_ × *m*_2_ image, *H* be a convolution (or blurring) operator, *η*_1_ be an additive Gaussian noise, and *f* be a degraded image which obeys the following formulation

$$f=Hu+\eta_{1}$$ (1)

Given *H* (the blurr operator), our objective is to restored *u* from *f*, which is called as de-convolution or de-blurring process. When *H* is the unit (identity) operator, recovering *u* from *f* is referred as Gaussian (additive) noise removal.

In practice, there are other types of noise as well such as multiplicative noise. It can also degrade an image. In recent years, many scientists have studied the issue of restoring images corrupted with multiplicative noise. For a computational formulation of such degradations, assume that an image *u* is defined on Ω ⊆ *R*^2^ with compact Lipschitz boundary that is *u*: Ω → *R*. The model of the contamination process of image *f* can be reformulated as follows

$$f=\left(Hu\right)\eta_{2}$$ (2)

where *H* is a linear and continuous blurring operator and *η*_2_ is a multiplicative noise which follows a Gamma distribution with mean one. Here, *u* is degraded by the blurring operator *H* to capture *f* > 0, and is then contaminated by the multiplicative noise *η*_2_. The degradation process leads to the multiplicative noise removal problem, when *H* is the identity operator. There are numerous applications for multiplicative noise reduction. For instance, magnetic field inhomogeneity in magnetic resonance imaging (MRI), speckle noise in synthetic aperture radar (SAR) images and in ultrasound. In these applications, *η*_2_ is supposed to follow some probability distributions. For example, the Rayleigh distribution is considered in ultrasound imaging, while the Gamma distribution is studied in SAR images. On the other hand, the contamination by multiplicative noise and blur occurs in many optical coherent imaging systems. Such non-linear image restoration problems can be transformed into the model given in Eq (2), where both multiplicative noise and blur appear.

### 1.1 Multiplicative Noise Removal

Images propagated by coherent imaging systems, for example, due to the coherent nature of the scattering phenomena, ultrasound imaging, laser and SAR imaging, inevitably come with multiplicative noise. The presence of this issue restrict us from studying valuable information of images, such as sharp edges, point target and textures, and hence multiplicative noise removal is a necessary pre-processing task for successful utilization of image processing tools involving detection, classification and image segmentation. Multiplicative noise seriously interrupts with fundamental tasks, such as object recognition or target detection, image segmentation and classification. Due to the coherent nature of the image acquisition systems, in the multiplicative noise models, the noise field is multiplied by the observed image, which is described by a probability density functions (non-Gaussian), with Gamma and Rayleigh being common models [16, 17].

Multiplicative noise is a non-Gaussian, signal independent and spatially dependent i.e variance is a function of signal amplitude. In the case of multiplicative noise, the variance of the noise is higher when an amplitude of the signal is higher. In other words, noise in bright regions has higher variations and could be interpreted as features in the given image. Hence multiplicative noise removal which is one of most complex images noise, is an important task when it comes to smoothing noise without degrading true image features. The popular despeckling methods include spatial, wavelet-based, non-local filtering and variational. In this work, we will focus on developing a new variational method for both multiplicative noise and blur removal. For more details see [18–30].

To the best of our knowledge, there exist several variational approaches devoted to the problem of multiplicative noise reduction. The first total variation based approach for multiplicative noise removal is the one introduced by Rudin et al. [23] which used a constrained optimization approach with two Lagrange multipliers. By applying a MAP-approximation, Aubert and Aujol [31] developed an energy functional whose minimization leads to the restored image to be recovered. Shi and Osher [32] utilize the fidelity term of the AA-model and used the *TV*(*logu*) filter instead of *TV*(*u*) and letting *w* = *logu*, they developed the total variation based strictly convex model (SO-model). Similarly, Bioucas and Figueiredo with SO-model, converted the multiplicative noise removal model into an additive one by taking logarithms and introduced Bayesian type variational model [33]. Steidl and Teuber [34] proposed a variational model which consist the I-divergence as data fidelity term and the TV-semi-norm as a regularization term. For multiplicative Gamma noise reduction, a variational model involving Curvelet coefficients was introduced in [35]. Most recent works include, both additive and multiplicative noise removal model by Chumchob et al. [36], a higher-order MRF based variational model for removing multiplicative noise presented by Chen et al. [37], speckle removal via higher-order TV based approach introduced by Feng et al. [16] and speckle noise removal via non-convex high total variation approach by Wu et al. [17] and so on.

### 1.2 Blur Reduction

There are some works dealing with joint multiplicative noise and blur removal; see [1, 2, 23, 38–42]. In this regard, both the RLO [23] and AA [31] models can be extended to handle the deblurring problem. Dong et al. [1] developed a convex variational model for recovering multiplicative noisy images with blur which consists a quadratic term, a MAP estimator based on Gamma noise and the total variational filter. The quadratic term is based on the statistical principle of the Gamma distribution noise. The uniqueness of the proposed model is assured under a mild condition, where the multiplicative noise follows a Gamma distribution. Recently, Zhao et al. [2] proposed a new convex minimization model for restoring blurred images with multiplicative noise, which consist the total variational penalty, the inverse of noise variance, the *l*_1_-norm of the data-fidelity term among the noise, observed image and image variables. Numerical outputs demonstrate that the considered model can tackle the blur and multiplicative noise reduction quite well. In [38], Wang et al. proposed on log-image domain a constrained image restoration model which is based on total variational filter and estimated a set of non-convex constraints by a set of convex constraints. The alternating direction method of multipliers was employed to handle the resulting minimization problem. Huang et al. [42] introduced a new variational approach by adding a Gaussian noise term in the functional to handle the multiplicative denoising and deblurring problem while used the alternating minimization method to find the minimizer of the energy functional efficiently.

### 1.3 Total Generalized Variation (TGV) Functional

Since solution of optimization problems with total variational filter is very effective for preserving sharp edges, corners and other fine details of an image. However, it has some disadvantages as well, most notably the so-called staircase effect that is the unwanted occurrence of edges. This fact arose from the assumption that the structure of the image is modeled as a function belonging to bounded variation space and hence it favors a piecewise constant function in bounded variation space. Under such an assumption, the total variation based model produces optimal results. As a matter of fact, staircase effect normally occurs since most of the natural images are not piecewise constant functions. To overcome the weakness of the total variation functional i.e the stair-casing effect, the modification of TV-model, which generalizes the differential order in regularization term, has attracted the more and more attentions of numerous researchers.

Recently, Bredies et al. [43] developed the total generalized variation (TGV) regularizer, which is assumed to be the generalization of the total variational filter. Total generalized variation consists and balances higher-order derivatives of *u*. Defining total generalized variation (TGV) according to Eq (3), one can prove that it constitutes a convex, proper, and weak lower semi-continuous regularizer on each *L*^*p*^(Ω) space, where 1 ≤ *p* ≤ ∞. TGV being a rotation and translation invariant, can be utilized as a penalty functional for variational imaging problems. Denoising and image restoration with TGV-filter often leads to piecewise polynomial intensities and sharp edges which efficiently tackle the blocky effect and produces better visual quality images than the traditional TV [44]. Therefore, the total generalized variation penalty has been extensively used in the variational models for deblurring, the reduction of Gaussian noise, multiplicative noise, and some other applications.

In this subsection, we give a brief introduction and summarize the results on total generalized variation (TGV) functional which are relevant for the present paper, and then proposed a new variational model for both multiplicative noise and blur removal based on it. First, redefine the TGV. For this purpose, consider Ω ⊂ ℜ^*d*^ is a non-empty, connected and open set. Remember that, *d* ∈ *Z*, *d* ≥ 1 is a fixed dimension space. Here, we choose *d* = 2 for the observed images. The TGV of order *g* and positive weights *γ* = (*γ*_0_, *γ*_1_, *γ*_2_, …*γ*_*g*−1_) can be reformulated as follows

$$TGV_{\gamma }^{g}\left(u\right)=sup\left\{\int_{\Omega }u\;div^{g}vdx|v\in C_{c}^{g}\left(\Omega ,Sym^{g}\left({ℜ}^{d}\right)\right),{∥div^{l}v∥}_{\infty }\leq \gamma_{l},l=0,1,2,...g-1\right\}$$ (3)

where *Sym*^*g*^(ℜ^*d*^) is the symmetric tensors space of order *g* with arguments in ℜ^*d*^ and $C_{c}^{g}\left(\Omega ,Sym^{g}\left({ℜ}^{d}\right)\right)$ represents compactly supported symmetric tensor field vector space and *sup*(⋆) denotes the least upper bound (l.u.b) of a set ⋆. When *g* = 2, *Sym*^2^(ℜ^*d*^) corresponds to all symmetric *d* × *d* matrices space and so on. Note that $TGV_{\gamma }^{g}\left(u\right)$ is a semi-norm that is zero for all polynomials of degree equal to or less than *g* − 1. Hence reconstruction with total generalized variation regularization results to images with piecewise polynomial intensities and hence is enable to preserve sharp edges well.

In this study, our focal point is the total generalized variation of second order that is ($TGV_{\gamma }^{2}\left(u\right)$). In this case, Eq (3) can be formulated as follows

$$TGV_{\gamma }^{2}\left(u\right)=sup\left\{\int_{\Omega }u\;div^{2}wdx|w\in C_{c}^{2}\left(\Omega ,Sym^{2}\left({ℜ}^{d}\right)\right),{∥div^{l}w∥}_{\infty }\leq \gamma_{l},l=0,1\right\}$$ (4)

We need discrete version of $TGV_{\gamma }^{2}\left(u\right)$, to analyze its properties. In this regard, the basic corresponding operators should be reviewed at the earlier stage [16]. First, the space $C_{c}^{2}\left(\Omega_{1},{ℜ}^{1}\right)$ is denoted by *U*, the space $C_{c}^{2}\left(\Omega_{1},{ℜ}^{2}\right)$ by *V* that is the elements of *υ* ∈ *V* are *υ*_1_ ∈ *U* and *υ*_2_ ∈ *U* and $C_{c}^{2}\left(\Omega_{1},S^{2\times 2}\right)$ by *W*. Similarly, the elements of *w* ∈ *W* are *w*^11^ ∈ *U*, *w*^12^ ∈ *U* and *w*^22^ ∈ *U*. Secondly, the first order forward and backward difference operators for *u* ∈ *U* are described as $\partial_{x}^{+}u,\partial_{y}^{+}u,\partial_{x}^{-}u\text{ and }\partial_{y}^{-}u$. Thirdly, the gradient, the symmetrized gradient and the divergence operator are reformulated as follows

$$\begin{array}{c} \nabla :U\to V,\nabla u=\left(\begin{array}{c} \partial_{x}^{+}u\\ \partial_{y}^{+}u \end{array}\right),\bar{\varepsilon }:V\to W,\bar{\varepsilon }\left(\upsilon \right)=\left(\begin{array}{cc} \partial_{x}^{-}\upsilon_{1} & \frac{1}{2}\left(\partial_{y}^{-}\upsilon_{1}+\partial_{x}^{-}\upsilon_{2}\right)\\ \frac{1}{2}\left(\partial_{y}^{-}\upsilon_{1}+\partial_{x}^{-}\upsilon_{2}\right) & \partial_{y}^{-}\upsilon_{2} \end{array}\right) \end{array}$$ (5)

$$\begin{array}{c} div_{1}:V\to U,div_{1}\left(\upsilon \right)=\partial_{x}^{-}\upsilon_{1}+\partial_{y}^{-}\upsilon_{2},div_{2}:W\to V,div_{2}\left(w\right)=\left(\begin{array}{c} \partial_{x}^{+}w^{11}+\partial_{y}^{+}w^{12}\\ \partial_{x}^{+}w^{12}+\partial_{y}^{+}w^{22} \end{array}\right) \end{array}$$ (6)

Recall that the above operators are adjoint to each other that is (*div*_1_)* = −∇ and ${\left(div_{2}\right)}^{*}=-\bar{\varepsilon }$. According to [45], the discretized form of $TGV_{\gamma }^{2}\left(u\right)$ can be represented as

$$TGV_{\gamma }^{2}\left(u\right)=\min_{\upsilon \in V}\gamma_{1}{∥\nabla u-\upsilon ∥}_{1}+\gamma_{0}{∥\bar{\varepsilon }\left(\upsilon \right)∥}_{1}$$ (7)

Here, the balance between the first and second order derivatives of *u* is provided by the definition in Eq (7). According to [17], the multiplicative noise removal with TGV regularization both can results to piecewise polynomial smoothness and can preserve edges of the images. See [16, 17, 43–45] for more details on TGV.

**Remark:** The bounded generalized variation space can be defined as

$$BGV^{g}\left(\Omega \right)=\left\{u\in L^{1}\left(\Omega \right):TGV_{\gamma }^{g}\left(u\right)<\infty \right\}{;|u|}_{BGV^{g}}={\left|u\right|}_{1}+TGV_{\gamma }^{g}\left(u\right)$$

where *BGV*^*g*^(Ω) which is independent of the weight vector *γ* is a Banach space. On this space $TGV_{\gamma }^{g}\left(u\right)$ is a semi-norm that is zero for all polynomials of degree up to *g* − 1. Thus recovering images with $TGV_{\gamma }^{g}\left(u\right)$ results to piecewise polynomial intensities and its convex property makes it numerically optimal.

### 1.4 Shearlet Transform

Labate et al. [46] proposed shearlet transform based on wavelet which yields nearly optimal approximation properties. Based on isotropic dilations, the well-known wavelet transform is able to identify singular points of signals. However, it has limited ability to describe the geometry of multi-dimensional functions, for example, the edge orientation, while depend on anisotropic dilation, Shearlet transform is a directional representation system that provides more geometrical image information [47]. Shearlets have been proven mathematically to demonstrate distributed discontinuities such as sharp edges better than wavelets and are a useful tool for edge characterization. In other words, the shearlet transform is a very effective tool for tackling the piecewise smooth images containing corners, edges and spikes etc. The shearlets transform can completely approximate the piecewise smooth images’ singular structures. Such property of shearlets is suitable particularly in image processing task since irregular structures and singularities carry important details in an observed image. For example, discontinuities in the intensity of an image show the presence of edges. We remark that shearlets play the role of like the gradient, surely it can be employed to indicate the presence of an edge.

Shearlet transforms are designed to encode anisotropic features such as singularities concentrated on lower dimensional embedded manifolds. To obtain optimal sparsity, shearlets are scaled according to a parabolic scaling law encoded in the parabolic scaling matrix *A*_*a*_, *a* > 0, and exhibit directionality by parameterizing slope encoded in the shear matrix *S*_*s*_, *s* ∈ *R*, defined by

$$A_{a}=\left(\begin{array}{cc} a & 0\\ 0 & \sqrt{a} \end{array}\right)\text{ and }S_{s}=\left(\begin{array}{cc} 1 & s\\ 0 & 1 \end{array}\right)$$

where, *A*_*a*_ is an anisotropic dilation operator and *S*_*s*_ is a shear operator. Hence the shearlet system {*ψ*_*ast*_: *a* ∈ *R*^+^, *s* ∈ *R*, *t* ∈ *R*^2^} based on three parameters namely *a* > 0, *s* ∈ *R* and *t* ∈ *R*^2^. *a* > 0 is the scale parameter measured the resolution level, *s* ∈ *R* is the shear parameter approximate the directionality, and *t* ∈ *R*^2^ is the translation parameter measured the position which is produced by using the operations of dilation, shear transformation and translation on *ψ* ∈ *L*^2^(*R*^2^):

$$\psi_{a,s,t}\left(x\right)={\left|detM_{a,s}\right|}^{\frac{-1}{2}}\psi \left(M_{a,s}^{-1}\left(x-t\right)\right)$$

where $M_{a,s}=(\begin{array}{cc} a & \sqrt{a}s\\ 0 & \sqrt{a} \end{array})=(\begin{array}{cc} 1 & s\\ 0 & 1 \end{array})(\begin{array}{cc} a & 0\\ 0 & \sqrt{a} \end{array})=S_{s}A_{a};a\in R^{+},s\in R$. Hence to each matrix *M*_*a*,*s*_ two distinct actions are associated that is the anisotropic dilation is produced by the matrix *A*_*a*_ and the non-expansive matrix *S*_*s*_ produced shearing. The corresponding continuous shearlet transform of a function *f* ∈ *L*^2^(*R*^2^) can be reformulated as

$${\text{SH}}_{\psi }\left(f\right)\left(a,s,t\right)=\langle f,\psi_{a,s,t}\rangle$$

where *a* > 0, *s* ∈ *R*, *t* ∈ *R*^2^ and the analyzing elements *ψ*_*a*,*s*,*t*_ called shearlets are defined above. The shearlet transform is invertible if *ψ* possesses the following property

$$\int_{R^{2}}\frac{|\hat{\psi }\left(w_{1},w_{2}\right){|}^{2}}{|w_{1}{|}^{2}}dw_{1}dw_{2}<\infty$$

where *w*_1_, *w*_2_ ∈ *R*^2^ and the Fourier transform of *ψ* is denoted by $\hat{\psi }$. Other directional representation systems, such as ridgelets [48], contourlets [49], curvelets [50], have links with shearlets. For example, shearlets and curvelets both produce good results in showing images with edges, while both have different spatial-frequency tilings. Shearlets and curvelets are relatively close to contourlets, which are developed in a purely discrete format. In this paper, we develop shearlets due to their theoretical relation to the multi-resolution analysis, directional sensitivity and availability of efficient implementation. From the perspective of computational complexity, the discrete shearlet needs $m_{1}^{2}logm_{1}$ FLOPS for an *m*_1_ × *m*_1_ image. We refer to [51, 52] for more details regarding the shearlet transform.

The aim of this work, is to develop a new variational model for removing multiplicative noise (MN) and blur. We incorporate the total generalized variation functional and the shearlet transform into the existing data fidelity term for restoring images, which leads to good recovering results. The TGV and shearlet transform-based variational model outperform the traditional total variation methods by minimizing the blocky effects, as such total generalized variation involves and balances higher order derivatives of the image and the shearlet filter show anisotropic features such as curves and edges well. The proposed energy functional is efficiently solved by the alternating direction method of multiplier (ADMM). Experimental results illustrate advantages of our proposed model, both visually and quantitatively in multiplicative noise and blur removal simultaneously comparing to other recent methods.

The outline of this paper is as follows. In section 2, we give a brief review of the image restoration models M1, M2 and M3 for recovering blurred images with multiplicative noise. Then a new variational model for joint multiplicative denoising and deblurring is proposed. Afterwards, in section 3, numerical method is employed to solve the proposed variational problem efficiently. Subsequently, section 4 shows the choice of the regularization parameters and experimental results. Section 5 concludes the paper.

## 2 Problem Formulation

In this section, we commence with a brief introduction of the three current total variation based models for multiplicative noise and blur removal, and then a new variational model is proposed for the same problem.

### 2.1 Review of Total Variation Based Image Restoration Models

Total variation based regularization models have been proven to be a very valuable technique for image restoration, and are applied in many practical applications. In this subsection, the following three current state of the art TV-based models for removing multiplicative noise and blur are briefly reviewed namely; (a) M1: a fast minimization method for blur and multiplicative noise removal in [38](2012), (b) M2: a convex variational model for restoring blurred images with multiplicative noise in [1](2013) and (c) M3: a new convex optimization model for multiplicative noise and blur removal in [2](2014).

#### 2.1.1 Wang-Model (M1)

In [38], an efficient algorithm is developed by Wang et al., for solving the total variation based optimization model to restore images from input multiplicative noisy and blurred images. They used logarithmic function to transform multiplicative noise and blurring problems into additive noisy image problems and then apply *l*_1_-norm to measure in the data fidelity term and the total variational filter to measure the regularization term. The alternating direction method of multipliers (ADMM) is employed to solve the associated optimization problem, which can be reformulated as

$$\begin{array}{c} \underset{u}{min}{∥\nabla u∥}_{1}\text{ subject to }u\in R^{n}\\ {∥logf-logHu∥}_{1}\leq \alpha ;\text{ where }logf=logHu+log\eta \end{array}$$ (8)

Herein, the number of pixels is denoted by *n*, *α* ∈ *R*^+^, measures the trade off between the fit to *f* and ∇: *R*^*n*^ → *R*^*n*^, the amount of regularization is a discrete form of the gradient ∇ and ‖•‖_1_ denotes the *l*_1_-norm. *H* is the blur operator and *η* is a noise.

#### 2.1.2 Dong-Model (M2)

A new variational model for restoration of blurred images with multiplicative noise is introduced by Dong et al. in [1], and then a primal-dual algorithm to tackle the minimization model in Eq (9) is employed. A strictly convex model is developed under a particular condition which assured the uniqueness of the solution and stability of the algorithm. For this purpose, a quadratic penalty function is added in the energy functional. Using Bayesian formula, the following energy functional is proposed for the above mentioned problem

$$\begin{array}{c} \underset{u\in \bar{S}\left(\Omega \right)}{inf}E_{H}\left(u\right)=\lambda \int_{\Omega }\left|\nabla u\right|dxdy+\alpha \int_{\Omega }{\left(\sqrt{\frac{Hu}{f}}-1\right)}^{2}dxdy+\int_{\Omega }\left(log\left(Hu\right)+\frac{f}{Hu}\right)dxdy \end{array}$$ (9)

where $H\in L(L^{2}\left(\Omega \right))$ is a linear (assumed to be a non-negative) and continuous blurring operator. *α* > 0, λ > 0 are the penalty and regularization parameters. In addition, a closed and convex set is defined as $\bar{S}\left(\Omega \right)=\left\{\vartheta \in BV\left(\Omega \right):\;\vartheta \geq 0\right\}$.

The detailed proof of existence and uniqueness for M2-model can be found in [1].

#### 2.1.3 Zhao-Model (M3)

Zhao et al. [2] introduced a new convex total variation based model for restoring images contaminated with multiplicative noise and blur. The main notion is to reformulate a blur and multiplicative noise equation such that both the image variable and noise variable are decoupled. As a result, the concluding energy functional involves the total variational filter, the term of variance of the inverse of noise, the *l*_1_-norm of the data fidelity term among the observed image, noise and image variables. The convex optimization model is given by

$$\min_{w,f}\frac{1}{2}{∥w-\mu e∥}_{2}^{2}+\alpha_{1}{∥Gw-Hf∥}_{1}+\alpha_{2}{∥Df∥}_{2}$$ (10)

where *α*_1_, *α*_2_ are two regularization parameters. *μ* denotes the average value of *w* and *e* is a vector of all ones. The first term in Eq (10) is to measure the variance of *w*. The second term is the fidelity term between the observed image, *f* and *w*. If *H* = *I*, then Eq (10) leads to an unconditional convex multiplicative noise removal model [2].

#### 2.1.4 The Proposed Model (M4)

In the real world problems, often the underlying image *f* is not only degraded by noise, but it is also by blurred. To handle such problems, variational approaches with total variation regularization have attracted the attention of many researchers which possess many desirable properties, most importantly the preservation of edges. However, the TV-filter also has some disadvantages, most notably the staircase effect. This undesired appearance of artifacts arises from the model assumption that an image contains piecewise constant areas, even in regions with smooth transitions of pixel values. To minimize the staircase effect, preserve sharp edges and other fine details of an image, we propose a new variational model which using total generalized variation $TGV_{\gamma }^{2}$ filter and shearlet transform as regularizers, for joint deblurring and multiplicative noise removal. This model obviously uses advantages of both $TGV_{\gamma }^{2}$ and shearlet transform, which leads to good restoration results, because $TGV_{\gamma }^{2}$ performs well for reducing the staircase effect, preserving sharp edges and textures while the Shearlet filter demonstrates anisotropic image features such as sharp edges and curves well. Hence using this model, reasonable improvement in PSNR values is obtained. The following functional is proposed for minimization

$$\begin{array}{c} \underset{u}{min}E_{H}\left(u\right)=\int_{\Omega }\left(log\left(Hu\right)+\frac{f}{Hu}\right)dxdy+\alpha \int_{\Omega }{\left(\sqrt{\frac{Hu}{f}}-1\right)}^{2}dxdy+\lambda \underset{j=0}{\sum^{N}}{\left|{\text{SH}}_{j}\left(u\right)\right|}_{1}+TGV_{\gamma }^{2}\left(u\right) \end{array}$$ (11)

Where the first two terms are the data fidelity terms. *H* is a linear and continuous blurring operator. *α* and λ are the regularization parameters. *u* is the restored image from the noisy data *f*. SH_*j*_(*u*) is the *j*^*th*^ sub-band of the shearlet transform of *u*. For computational implementation purpose, the fast finite shearlet transform (FFST) is adopted [52], in which the restoration is based on the wavelet functions and Meyer scaling. We use the second order $TGV_{\gamma }^{2}\left(u\right)$ as a regularizer. Moreover, all the band wise discrete shearlet transforms can be computed fastly using the fast Fourier transform (FFT) and the discrete inverse Fourier transform (IFT). The discrete shearlets contain a Parseval frame of finite Euclidean space while the inversion of the shearlet transform can be simply performed by employing the adjoint transform. Let the FFT of the discrete 2*D*-scaling function is denoted by *H*_1_ and *H*_*j*_(*j* ≥ 2) be those of the discrete shearlets. Let $MAT:C^{n\times n}\to C^{n^{2}}$ and $VEC:C^{n\times n}\to C^{n^{2}}$ be the matricizing and vectorizing functions. Therefore, we have

$${\text{SH}}_{j}\left(MAT\left(u\right)\right)=F^{-1}\left(H_{j}.*F\left(MAT\left(u\right)\right)\right)=F^{-1}(H_{j}*MAT\left(u\right)$$

where * and.* denote convolution and componentwise multiplication. The above equation in vector form is given by

$${\text{SH}}_{j}\left(u\right)=VEC\left({\text{SH}}_{j}\left(u\right)\right)=F^{*}diag\left(VEC\left(H_{j}\right)\right)Fu=M_{H_{j}}u$$

where $M_{H_{j}}=F^{*}diag(VEC(H_{j}))F$, and diag is defined as

$$diag:C^{N}\to C^{N\times N},\;diag{\left(u\right)}_{hj}=u_{h}\delta_{hj}$$

where *δ*_*hj*_ = 0; if *h* ≠ *j* and *δ*_*hh*_ = 1.

With the new formulation of $TGV_{\gamma }^{2}\left(u\right)$ as defined in Eq (7), the proposed model (11) can be reformulated as

$$\begin{array}{c} \underset{u,p}{min}E_{H}\left(u\right)=\int_{\Omega }\left(log\left(Hu\right)+\frac{f}{Hu}\right)dxdy+\alpha \int_{\Omega }{\left(\sqrt{\frac{Hu}{f}}-1\right)}^{2}dxdy+\lambda \underset{j=0}{\sum^{N}}{\left|{\text{SH}}_{j}\left(u\right)\right|}_{1}\\ +\gamma_{1}{∥\nabla u-p∥}_{1}+\gamma_{0}{∥\bar{\varepsilon }\left(p\right)∥}_{1} \end{array}$$ (12)

For a numerical realization, the discrete version of model (12) reads as follows

$$\begin{array}{c} \underset{u,p}{min}E_{H}\left(u\right)=\langle log\left(Hu\right),1\rangle +\langle \frac{f}{Hu},1\rangle +\alpha {∥{\left(\sqrt{\frac{Hu}{f}}-1\right)}^{2}∥}_{2}^{2}+\lambda \underset{j=0}{\sum^{N}}{\left|{\text{SH}}_{j}\left(u\right)\right|}_{1}\\ +\gamma_{1}{∥\nabla u-p∥}_{1}+\gamma_{0}{∥\bar{\varepsilon }\left(p\right)∥}_{1} \end{array}$$ (13)

where, the vector innner product $\langle u,v\rangle ={\underset{i=1}{\Sigma }}^{n}u_{i}v_{i}$ is used, and ‖.‖_2_ shows the *l*^2^-vector norm.

#### 2.1.5 Existence and Uniqueness of the Solution of the Proposed Model (M4)

Based on the properties of TGV (which is a convex, proper and weak lower semi-continuous regularizer), shearlet transform, fidelity term and the space of bounded generalized variation, we can prove the existence and uniqueness of the M4-model given in Eq (11).

**Coercivity assumption** [16, 53]: For any sequence {*u*^*n*^} in *L*^*q*^(Ω), such that $q\leq \frac{d}{d-1}\leq 2$ for *d* = 2, it follows that

$$∥Qu^{n}{∥}_{q}\to \infty \text{and}\{∥\left(I-Q\right)u^{n}{∥}_{q}\}\text{ bounded}\Rightarrow G\left(u^{n}\right)\to \infty$$ (14)

where $G\left(u^{n}\right)=\langle log\left(Hu^{n}\right),1\rangle +\langle \frac{f}{Hu^{n}},1\rangle +\alpha ∥{\left(\sqrt{\frac{Hu^{n}}{f}}-1\right)}^{2}{∥}_{2}^{2}$ with $\alpha \geq \frac{2\sqrt{6}}{9}$. Then there exists at least one minimizer for Eq (11).

According to the proposition-1 in [16] and the coercivity assumption Eq (14) being satisfied, together with proper, convex and lower semi-continuous G(u). It implies that there is a solution to Eq (11) which can be proved along similar lines to Ref. [1, 16]. The solution is unique due to each term in *E*_*H*_(*u*) being convex [1, 16, 43, 52, 53].

In the next section, we discuss how to employ the alternating direction method of multipliers, to solve the proposed model (M4).

## 3 Numerical Implementation

Let us briefly review the ADMM method for linearly constrained problems which solves the model in the form of

$$\begin{array}{c} \underset{r,s}{min}f_{1}\left(r\right)+f_{2}\left(s\right)\text{ subject to }Br+Cs=d \end{array}$$ (15)

Then, the augmented Lagrangian function is $L\left(r,s,t\right)=f_{1}\left(r\right)+f_{2}\left(s\right)+\frac{\beta }{2}{∥Br+Cs-d-t∥}_{2}^{2}$, where *t* is the scaled Lagrange multiplier and *β* > 0 is a parameter. The algorithm of ADMM starts from *s*^(0)^ = 0, *t*^(0)^ = 0 and iterates

$$\begin{array}{c} \left(i\right)\;r^{\left(n+1\right)}=\underset{r}{argmin}L\left(r,s^{\left(n\right)},t^{n}\right);\\ \left(ii\right)\;s^{\left(n+1\right)}=\underset{s}{argmin}L\left(r^{\left(n+1\right)},s,t^{\left(n\right)}\right);\\ \left(iii\right)\;t^{\left(n+1\right)}=t^{\left(n\right)}+\beta \left(d-\left(Br^{\left(n+1\right)}+Cs^{\left(n+1\right)}\right)\right). \end{array}$$ (16)

For the ADMM scheme, the convergence proofs and its variants can be followed from [38, 54–56].

### 3.1 ADMM Implementation

For each *l*_1_-term, we assigned one quadratic penalty term and one auxiliary variable for the implementation of ADMM algorithm to solve the optimization problem Eq (11). Particularly, we introduce auxiliary variables *x*_*j*_, *j* = 1, 2, …, *N*,

$$z=\left(\begin{array}{cc} z_{1} & z_{3}\\ z_{3} & z_{2} \end{array}\right)\in W;y=\left(\begin{array}{c} y_{1}\\ y_{2} \end{array}\right)\in V$$

such that Eq (13) may be reformulated equivalently as follows

$$\begin{array}{c} \underset{u,p,x_{j},y,z}{min}\langle log\left(Hu\right),1\rangle +\langle \frac{f}{Hu},1\rangle +\alpha ∥{\left(\sqrt{\frac{Hu}{f}}-1\right)}^{2}{∥}_{2}^{2}+\lambda \sum_{j=0}^{N}|x_{j}{|}_{1}+\gamma_{1}{∥y∥}_{1}+\gamma_{0}{∥z∥}_{1}\\ \text{ subject to }x_{j}={\text{SH}}_{j}\left(u\right),y=\nabla u-p,z=\bar{\varepsilon }\left(p\right) \end{array}$$ (17)

Where the absolute values of all components’ sum in *x*_*j*_ is denoted by ‖*x*_*j*_‖_1_, while the sum of *l*_2_-norms of all 2 × 1 vectors and 2 × 2 matrices is denoted by (‖*y*‖_1_, ‖*z*‖_1_).

After the implementation of ADMM, we formulate the following algorithm given as

$$\begin{array}{c} x_{j}^{\left(n+1\right)}=\underset{x_{j}}{argmin}|x_{j}{|}_{1}+\frac{\mu_{1}}{2}{∥x_{j}-{\text{SH}}_{j}\left(u^{\left(n\right)}\right)-\tilde{x_{j}^{\left(n\right)}}∥}_{2}^{2};j=1,2,..,N\\ y^{\left(n+1\right)}=\underset{y}{argmin}{\left||y|\right|}_{1}+\frac{\mu_{2}}{2}{∥y-\left(\nabla u^{\left(n\right)}-p^{\left(n\right)}\right)-\tilde{y^{\left(n\right)}}∥}_{2}^{2};\\ z^{\left(n+1\right)}=\underset{z}{argmin}{\left||z|\right|}_{1}+\frac{\mu_{3}}{2}{∥z-\bar{\varepsilon }\left(p^{\left(n\right)}\right)-\tilde{z^{\left(n\right)}}∥}_{2}^{2};\\ \left(u^{\left(n+1\right)},p^{\left(n+1\right)}\right)=\underset{u,p}{argmin}\frac{\lambda \mu_{1}}{2}\sum_{j=1}^{N}∥x_{j}^{\left(n+1\right)}-{\text{SH}}_{j}\left(u\right)-\tilde{x_{j}^{\left(n\right)}}∥+\frac{\gamma_{1}\mu_{2}}{2}{∥y^{\left(n+1\right)}-\left(\nabla u^{\left(n\right)}-p\right)-\tilde{y^{\left(n\right)}}∥}_{2}^{2}\\ +\frac{\gamma_{0}\mu_{3}}{2}{∥z^{\left(n+1\right)}-\bar{\varepsilon }\left(p\right)-\tilde{z^{\left(n\right)}}∥}_{2}^{2}+\langle log\left(Hu\right),1\rangle +\langle \frac{f}{Hu},1\rangle +\alpha ∥{\left(\sqrt{\frac{Hu}{f}}-1\right)}^{2}{∥}_{2}^{2}\\ {\tilde{x_{j}}}^{\left(n+1\right)}={\tilde{x_{j}}}^{\left(n\right)}+\mu \left({\text{SH}}_{j}\left(u^{\left(n+1\right)}\right)-x_{j}^{\left(n+1\right)}\right);j=1,2,...,N\\ {\tilde{y}}^{\left(n+1\right)}={\tilde{y}}^{\left(n\right)}+\mu \left(\nabla u^{\left(n+1\right)}-p^{\left(n+1\right)}-y^{\left(n+1\right)}\right);\\ {\tilde{z}}^{\left(n+1\right)}={\tilde{z}}^{\left(n\right)}+\mu \left(\bar{\varepsilon }\left(p^{\left(n+1\right)}\right)-z^{\left(n+1\right)}\right). \end{array}$$ (18)

The first three sub-problems are solved by shrinkage explicitly. The x-subproblem and y-problem can be solved by

$$\begin{array}{c} x_{j}^{\left(n+1\right)}=shrink_{1}\left({\text{SH}}_{j}\left(u^{\left(n\right)}\right)+{\tilde{x_{j}}}^{\left(n\right)},\frac{1}{\mu_{1}}\right);\text{for }j=1,2,...,N;\text{ where}\\ shrink_{1}\left(\nu ,\sigma \right)=sgn\left(\nu \right).*max\left(|\nu |-\sigma ,0\right) \end{array}$$ (19)

$$\begin{array}{c} y^{\left(n+1\right)}\left(l\right)=shrink_{2}\left(\nabla u^{\left(n\right)}\left(l\right)-p^{\left(n\right)}\left(l\right)+{\tilde{y}}^{\left(n\right)}\left(l\right),\frac{1}{\mu_{2}}\right),l\in \Omega \end{array}$$ (20)

where the component of *y*^(*n*+1)^(*l*) located at *l* ∈ Ω is denoted by *y*^(*n*+1)^(*l*) ∈ *R*^2^, and the shrinkage operator *shrink*_2_ can be formulated as follows

$$shrink_{2}\left(a,\mu \right)=\left\{\begin{array}{cc} 0 & \;a=0,\\ {(∥a∥}_{2}-\mu )\frac{a}{{∥a∥}_{2}} & \;a\neq 0 \end{array}\right.$$

Similarly, solution for the z-problem is formulated as

$$\begin{array}{c} z^{\left(n+1\right)}\left(l\right)=shrink_{F}\left(\bar{\varepsilon }\left(p^{\left(n\right)}\right)\left(l\right)+{\tilde{z}}^{\left(n\right)}\left(l\right),\frac{1}{\mu_{3}}\right),l\in \Omega \end{array}$$ (21)

where the component of *z*^(*n*+1)^ corresponding to the pixel *l* ∈ Ω is given by *z*^(*n*+1)^(*l*) ∈ *S*^2×2^ and

$$shrink_{F}\left(f,\mu \right)=\left\{\begin{array}{cc} 0 & \;f=0,\\ {(∥f∥}_{F}-\mu )\frac{f}{{∥f∥}_{F}} & \;f\neq 0 \end{array}\right.$$

where 0 is a square null matrix and the Frobenius norm of a matrix is denoted by ‖⋆‖_*F*_.

To summarize, the numerical algorithm is done in the following steps

**Algorithm 1** $TGV_{\gamma }^{2}\left(u\right)$ and Shearlet Transform Based Image Restoration by ADMM

1: **procedure** Input (*γ*_0_, *γ*_1_, *β*, λ, *μ*_*j*_, *μ*, j = 1,2,3.)

2: Initialize $u^{0},p_{1}^{0},p_{2}^{0},x_{j}^{0},{\tilde{x_{j}}}^{0}$,j = 1,2,$z_{j}^{0},{\tilde{z_{j}}}^{0}$,j = 1,2,3.

3: Computing *x*^(*n*+1)^, *y*^(*n*+1)^ and *z*^(*n*+1)^ by Eqs (19), (20) and (21) respectively.

4: Calculate $u^{\left(n+1\right)},p_{1}^{\left(n+1\right)}\text{ and }p_{2}^{\left(n+1\right)}$ from

$$\begin{array}{c} \left(u^{\left(n+1\right)},p^{\left(n+1\right)}\right)=\underset{u,p}{argmin}\frac{\lambda \mu_{1}}{2}\sum_{j=1}^{N}∥x_{j}^{\left(n+1\right)}-{\text{SH}}_{j}\left(u\right)-{\tilde{x_{j}}}^{\left(n\right)}∥+\frac{\gamma_{1}\mu_{2}}{2}{∥y^{\left(n+1\right)}-\left(\nabla u^{\left(n\right)}-p\right)-\tilde{y^{\left(n\right)}}∥}_{2}^{2}\\ +\frac{\gamma_{0}\mu_{3}}{2}{∥z^{\left(n+1\right)}-\bar{\varepsilon }\left(p\right)-\tilde{z^{\left(n\right)}}∥}_{2}^{2}+\langle log\left(Hu\right),1\rangle +\langle \frac{f}{Hu},1\rangle +\alpha ∥{\left(\sqrt{\frac{Hu}{f}}-1\right)}^{2}{∥}_{2}^{2} \end{array}$$ (22)

5: Calculate ${\tilde{x_{j}}}^{\left(n+1\right)},{\tilde{y_{j}}}^{\left(n+1\right)}\text{and}{\tilde{z_{j}}}^{\left(n+1\right)}$ for *n* = 0, 1, 2, ….. by

$$\begin{array}{c} {\tilde{x_{j}}}^{\left(n+1\right)}=\mu \left({\text{SH}}_{j}\left(u^{\left(n+1\right)}\right)-x_{j}^{\left(n+1\right)}\right)+{\tilde{x_{j}}}^{\left(n\right)};\text{for }j=1,2,...,N.\\ {\tilde{y}}^{\left(n+1\right)}=\mu \left(\nabla_{j}u^{\left(n+1\right)}-p_{j}^{\left(n+1\right)}-y_{j}^{\left(n+1\right)}\right)+{\tilde{y}}^{\left(n\right)};\text{for }j=1,2.\\ {\tilde{z}}^{\left(n+1\right)}=\mu \left(\bar{\varepsilon }{\left(p^{\left(n+1\right)}\right)}_{j}-z_{j}^{\left(n+1\right)}\right)+{\tilde{z}}^{\left(n\right)};\text{for }j=1,2,3. \end{array}$$ (23)

6: Output *u*^(*n*+1)^, If the stopping criteria is satisfied.

7: **end procedure**

## 4 Experimental Results

In this section, some numerical tests are presented to show the performance of our proposed model (M4) for restoring images contaminated with multiplicative noise and blur simultaneously. The results are compared with three models namely, (a)M1-model [38] (b) M2-model [1] (c) M3-model [2]. The proposed ADMM-algorithm is tested on different images of size (256^2^). In this work, two quality tools are used to obtain the restoration results. The first one is the peak signal to noise ratio (PSNR) and the other one is the structural similarity index (SSIM). These measures are given by

$$\text{PSNR}=10*log_{10}\left[\frac{N^{2}m_{1}m_{2}}{{\underset{i=1}{\Sigma }}^{m_{1}}{\underset{i=1}{\Sigma }}^{m_{2}}{\left(\hat{u}(i,j)-u(i,j)\right)}^{2}}\right]$$ (24)

$$\text{SSIM}=\frac{\left(2\mu_{\hat{u}}\mu_{u}\right)\left(2\sigma_{\hat{u}u}+a_{2}\right)}{\left(\mu_{\hat{u}}^{2}+\mu_{u}^{2}+a_{1}\right)\left(\sigma_{\hat{u}}^{2}+\sigma_{u}^{2}+a_{2}\right)}$$ (25)

where the maximal gray level of the image is denoted by *N*, *u* is the clean image, $\hat{u}$ is the restored image, $\mu_{\hat{u}}$, *μ*_*u*_, $\sigma_{\hat{u}}^{2}$, $\sigma_{u}^{2}$, $\sigma_{\hat{u}u}$, *a*_1_, *a*_2_ are the average values of $\hat{u},u$, variance of $\hat{u},u$, covariance of $\hat{u},u$ and two small positive constants respectively. The SSIM value range lies in [0, 1] with 1 for the optimal quality. The relative error (RelErr) of the image $\hat{u}$ is given by

$$\text{RelErr}=\frac{∥\hat{u}{-u∥}_{2}^{2}}{{∥u∥}_{2}^{2}}$$ (26)

All simulations listed here are employed in MATLAB-R2013a and all tests were carried out on Intel(R) Core(TM) i3-4160 CPU @360GHz 3.60 GHz, 8.00 Gb of RAM and 64-bit operating system.

### 4.1 Choosing the Regularization Parameters

**Fig 1:**
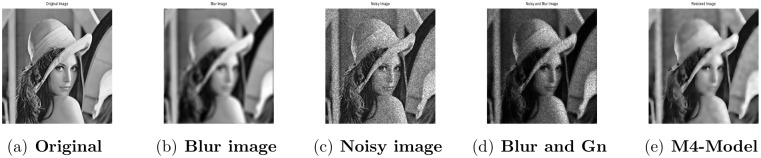
Performance of M4-model on Lena image. (a) original image; (b) blur image with (fspecial(‘disk’,5)); (c) noisy image with gamma noise variance 0.03; (d) the clean image contaminated by motion blur and the Gamma noise (Gn) with variance 0.03; (e) the restored image by our proposed model (M4).

**Fig 2:**
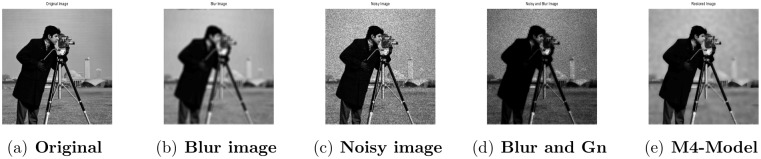
Performance of M4-model on Cameraman image. (a) original image; (b) blurry image with (fspecial(‘gaussian’,7,5)); (c) gamma noise variance is 0.03; (d) the given image degraded by motion blur and the Gamma noise (Gn) with variance 0.03; (e) the image restored by our proposed model (M4).

**Fig 3:**
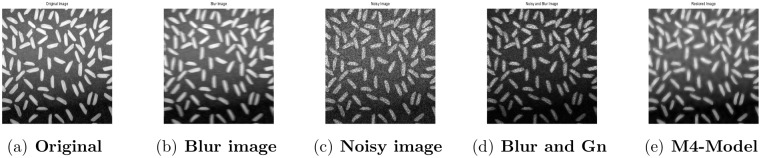
Performance of M4-model on Rice image. (a) original image; (b) blur image with (fspecial(‘motion’,7)); (c) gamma noise variance is 0.03; (d) the given image degraded by the Gamma noise (Gn) with variance 0.03 and motion blur; (e) the output image by M4-model.

**Fig 4:**
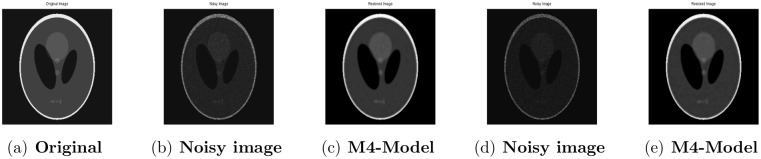
Performance of M4-model on Phantom image. (a) clean image; (b) degraded image with L = 10; (c) the restored image by our proposed model; (d) the observed image corrupted with L = 6 by multiplicative noise; (e) the recovered image by our proposed model (M4).

**Fig 5:**
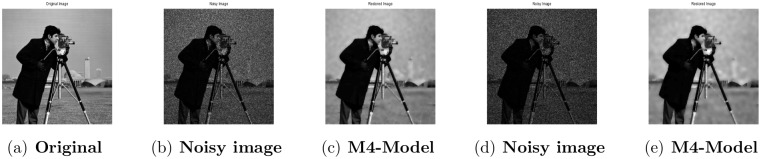
Performance of M4-model on Cameraman image. (a) original image; (b) degraded image with L = 10; (c) the restored image by our proposed model; (d) the observed image corrupted with L = 6; (e) the restored image by our proposed model (M4).

**Fig 6:**
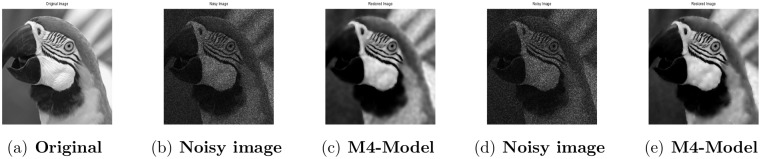
Performance of M4-model on Parrot image. (a) observed image; (b) noisy image with L = 10; (c) the restored image by our proposed model; (d) the observed image corrupted with L = 6; (e) the restored image by our proposed model (M4).

**Fig 7:**
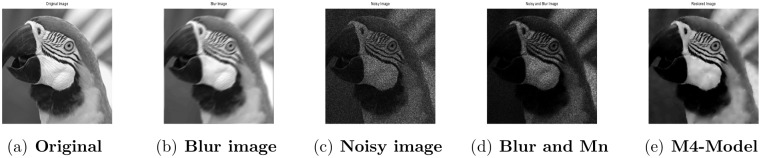
Performance of M4-model on Parrot image. (a) original image; (b) blur image with (fspecial(‘motion’,5,30)); (c) corrupted image with L = 10; (d) the original image contaminated by multiplicative noise with L = 10 and motion blur; (e) the recovered image by M4-model.

**Fig 8:**
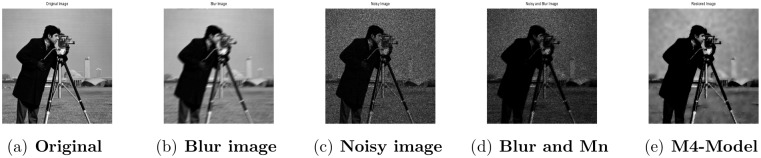
Performance of M4-model on Cameraman image. (a) original image; (b) blur image with (fspecial(‘motion’,5,30)); (c) degraded image with L = 10; (d) the clean image degraded by motion blur and the multiplicative noise with L = 10; (e) the restored image by the M4-model.

**Fig 9:**
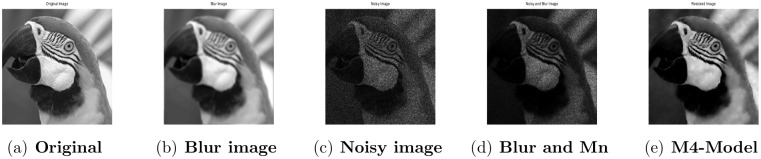
Performance of M4-model on Parrot image. (a) original image; (b) blur image with (fspecial(‘gaussian’,[7,7,2)); (c) contaminated image with L = 10; (d) the given image contaminated by motion blur and the multiplicative noise with L = 10; (e) the restored image by our proposed model (M4).

**Fig 10:**
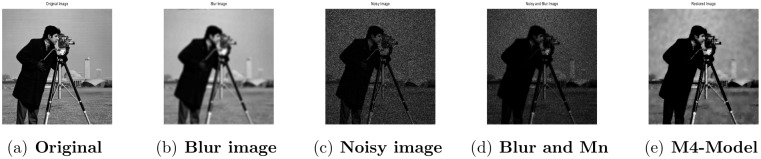
Performance of M4-model on Cameraman image. (a) original image; (b) blur image with (fspecial(‘gaussian’,[7,7],2)); (c) degraded image with L = 10; (d) the original image degraded by multiplicative noise with L = 10 and motion blur; (e) the restored image by our proposed model (M4).

**Fig 11:**
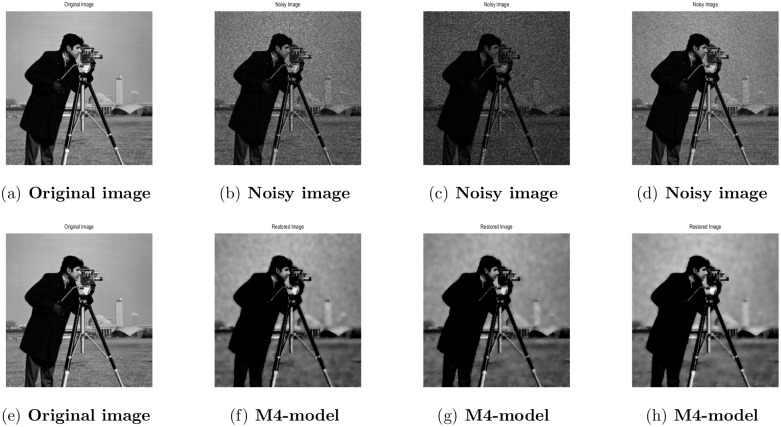
Performance of M4-model on Cameraman image. (a) clean image; (b) the observed image contaminated by the Gaussian noise; (c)the observed image contaminated by the Gamma noise; (d) the observed image contaminated by the Rayleigh noise; (e) original image; (f) the recovered image by our proposed model (M4); (g) restoration by our proposed model (M4); (h) the restored image by M4-model.

**Fig 12:**
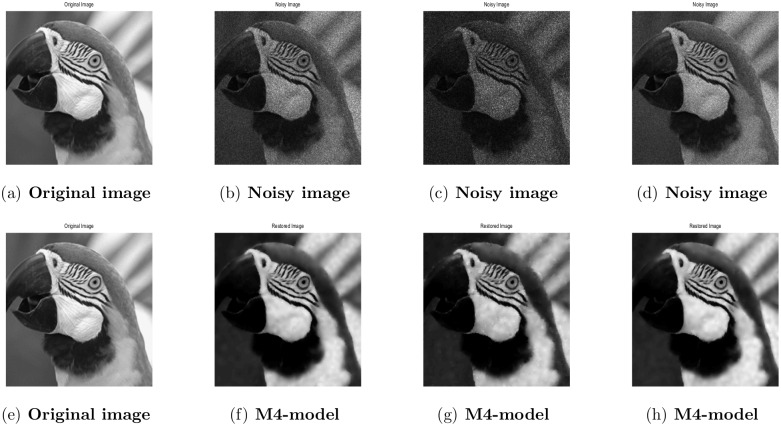
Performance of M4-model on Parrot image. (a) original image; (b) the observed image contaminated by the Gaussian noise; (c)the observed image contaminated by the Gamma noise; (d) the observed image contaminated by the Rayleigh noise; (e) original image; (f) the recovered image by M4-model; (g) restoration by M4-model; (h) the recovered image by M4-model.

**Fig 13:**
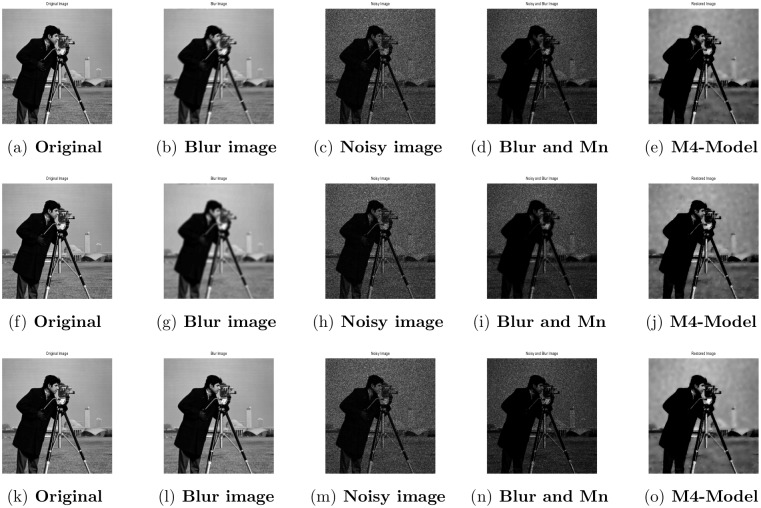
Performance of M4-model on Cameraman image. (a),(f),(k) original image; (b),(g),(l) blur image with (fspecial(‘motion’,5,30)), (fspecial(‘gaussian’,[7,7],2)), (psfMoffat(‘motion’,[7,7],1,5)); (c),(h),(m) degraded image with L = 10; (d),(i),(n) the clean image contaminated by motion blur, gaussian blur, Moffat and the Gamma noise with (L = 10); (e),(j),(o) the restored result by the M4-model.

**Fig 14:**
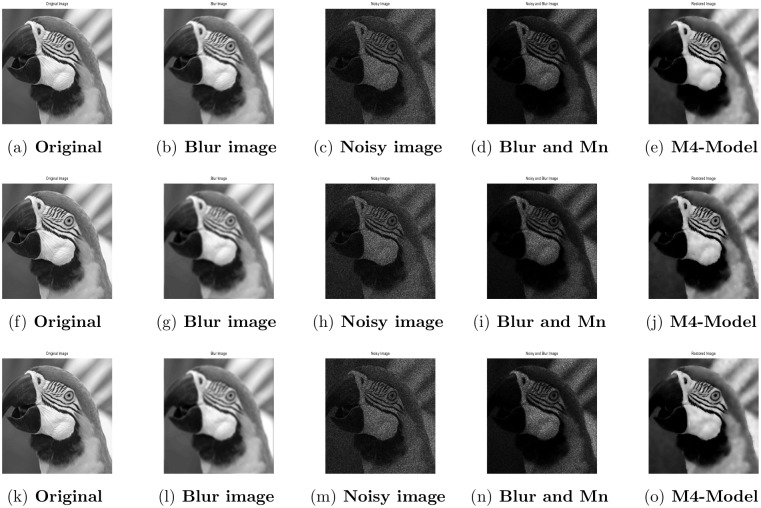
Performance of M4-model on Parrot image. (a),(f),(k) original image; (b),(g),(l) blur image with (fspecial(‘motion’,5,30)), (fspecial(‘gaussian’,[7,7],2)), (psfMoffat(‘motion’,[7,7],1,5)); (c),(h),(m) noisy image with L = 10; (d),(i),(n) the observed image degraded by motion blur, gaussian blur, Moffat and the Gamma noise with (L = 10); (e),(j),(o) the restored image by our proposed model (M4).

Like the total variation based models, the M4-model need a proper balance between the data fitting term and the regularization term. We set the values of regularization parameter λ and penalty parameter *α* empirically. Indeed, better restoration results may be obtained by performing optimal tuning of the parameters. The restoration results are promising already under the following adjusted and tuned parameter settings. For images, the choice of λ ∈ [1.5, 10] and $\alpha \geq \frac{2\sqrt{6}}{9}$ while number of iterations are considered to be in the range [100, 700] for images according to noise variance 0.01, 0.03 and L = 6, 10. Upon these ranges, better restoration results could be achieved with improved PSNR, SSIM and RelErr results. Results are presented in Tables 1–7 and Figs 1–14.

**Table 1:** Restoration models (M1 and M4) evaluated using PSNR and RelErr. Recall that the PSNR and RelErr of M1-model are reported in [38].

Image	Noise	Model	Motion blur		Gaussian blur		Disk blur	
			PSNR	RelErr	PSNR	RelErr	PSNR	RelErr
Lena	0.01	M1	25.02	0.1251	24.45	0.1319	22.82	0.1495
		M4	26.83	0.074	25.38	0.076	24.33	0.086
	0.03	M1	23.92	0.1569	24.22	0.1548	22.88	0.1706
		M4	24.35	0.086	25.13	0.088	24.99	0.089
Cameraman	0.01	M1	23.61	0.1333	22.84	0.1370	22.49	0.1582
		M4	25.24	0.083	25.16	0.083	25.89	0.08
	0.03	M1	22.58	0.1546	22.46	0.1599	21.74	0.1821
		M4	24.83	0.077	25.16	0.083	24.89	0.092
Rice	0.01	M1	26.22	0.1348	23.94	0.1539	23.15	0.1644
		M4	26.48	0.10	24.70	0.09	24.65	0.099
	0.03	M1	24.47	0.1656	23.08	0.1816	22.84	0.1945
		M4	25.61	0.100	24.69	0.099	24.52	0.101

**Table 2:** The number of iterations and time (in seconds) for distinct blurs and gamma noise levels showed in [38] using M1-model for ADM scheme and the number of ADMM iterations and time (in seconds) for different blurs and gamma noise levels reported using our model-M4.

Image	Noise	Model	Motion blur		Gaussian blur		Disk blur	
			Iteration	Time	Iteration	Time	Iteration	Time
Lena	0.01	M1	17	2.19	11	1.43	11	1.16
		M4	400	84.19	300	63.79	300	62.34
	0.03	M1	35	8.39	14	1.52	12	1.34
		M4	400	85.71	400	85.19	200	42.14
Cameraman	0.01	M1	18	1.23	42	2.78	11	1.06
		M4	400	84.98	400	85.15	400	86.18
	0.03	M1	20	1.43	12	1.52	11	1.14
		M4	400	86.62	400	88.75	200	43.01
Rice	0.01	M1	14	4.67	11	1.44	11	1.65
		M4	200	43.31	400	86.34	400	86.36
	0.03	M1	14	4.13	11	1.52	12	1.33
		M4	400	86.33	400	85.94	400	86.36

**Table 3:** Performance of M4-model using SSIM on Lena, Cameraman and Rice images.

Image	Noise	Model	Motion blur	Gaussian blur	Disk blur
			SSIM	SSIM	SSIM
Lena	0.01	M4	0.8345	0.8206	0.7924
	0.03	M4	0.7940	0.7897	0.7825
Cameraman	0.01	M4	0.8093	0.8075	0.8227
	0.03	M4	0.8224	0.8075	0.7875
Rice	0.01	M4	0.7365	0.7525	0.7516
	0.03	M4	0.7501	0.7515	0.7470

**Table 4:** Restoration models (M2 and M4) evaluated using PSNR, number of iterations and time. Recall that the PSNR, number of iterations and time of M2-model are reported in [1].

Image	Noise	Model	L = 10			L = 6		
			PSNR	Iter.	Time	PSNR	Iter.	Time
Phantom	L = 10,6	M2	30.44	132	6.09	28.05	174	8.24
		M4	31.41	173	7.05	29.43	167	6.83
Cameraman	L = 10,6	M2	25.01	162	8.50	28.05	174	8.24
		M4	25.75	300	66.77	28.20	300	65.22
Parrot	L = 10,6	M2	25.47	179	9.19	24.21	219	11.70
		M4	26.01	100	39.83	25.06	300	120.90

**Table 5:** Restoration models (M2 and M4) for deblurring with denoising evaluated using PSNR, number of iterations and CPU-time in seconds. Recall that the PSNR, number of iterations and CPU-time in seconds of M2-model are reported in [1].

Image	Noise	Model	Motion Blur			Gaussian blur		
			PSNR	Iter.	Time	PSNR	Iter.	Time
Phantom	L = 10	M2	24.68	182	84.78	22.59	331	152.85
		M4	25.05	300	110.30	23.97	300	109.23
Cameraman	L = 10	M2	22.99	200	91.93	21.85	293	119.09
		M4	24.67	300	128.09	22.96	300	128.19
Parrot	L = 10	M2	23.18	216	100.86	22.08	223	103.92
		M4	24.09	500	210.75	23.65	500	206.81

**Table 6:** Restoration results of M3-model and M4-model by evaluating PSNR, a no: of iterations and CPU-time in seconds. Recall that the PSNR, a no: of iterations and CPU-time in seconds of M3-model are displayed in [2].

Image	Noise	M3-Model			M4-Model		
		PSNR	Iteration	Time	PSNR	Iteration	Time
Cameraman	Gaussian	26.86	300	9.0	27.56	100	41.53
	Gamma	24.39	410	14.0	25.14	200	86.33
	Rayleigh	21.94	87	3.0	23.15	200	82.61
Parrot	Gaussian	27.21	290	10.86	28.85	200	80.99
	Gamma	24.75	331	11.0	25.69	300	125.39
	Rayleigh	20.74	86	3.0	23.29	200	81.35

**Table 7:** Restoration results of M3 and M4 for multiplicative deblurring, evaluated using PSNR, number of iterations and CPU-time in seconds. Recall that the PSNR, no. of iterations and CPU-time in seconds of M3-model are shown in [2].

Image	Blurs	Noise	M3-Model			M4-Model		
			PSNR	Iter.	Time	PSNR	Iter.	Time
Cameraman	Motion	L = 10	23.35	93.0	4.0	25.08	300	132.97
	Gaussian		22.42	35.0	2.0	24.21	300	136.61
	Moffat		24.10	52.0	2.0	25.69	300	144.94
Parrot	Motion	L = 10	23.18	106.0	5.0	24.32	300	123.33
	Gaussian		21.33	25.0	1.0	23.06	300	118.98
	Moffat		24.42	73.0	3.0	25.94	300	119.32

In the next subsection, we discuss the simulation results below, to demonstrate the effectiveness and feasibility of the M4-model. The experimental results of our M4-model are compared with those of M1-model, M2-model and M3-model.

### 4.2 Comparison of M4-Model with M1-Model

In this subsection, we report some numerical tests to analyze the performance of our M4-model for restoring images which are degraded by blur and multiplicative noise simultaneously. Here, the proposed model-M4 is compared with the one already discussed in [38] (M1-model). For this purpose, the restoration results of 256 × 256 gray scale images ‘Lena’, ‘Rice’ and ‘Cameraman’, which are already illustrated in [38] are considered. In the experiments, the clean image *u* is corrupted by a motion blur (Mat-lab function is, “fspecial(’motion’,7)”), a Gaussian blur (Mat-lab function is, “fspecial(’gaussian’,7,5)”) and a disk blur (Mat-lab function is, “fspecial(’disk’,5)”)) respectively and different levels of multiplicative gamma noise with variance 0.01 and 0.03. The PSNR, RelErr, SSIM of restored images, the required computational time and the number of iterations are displayed in Tables 1 and 2. From comparing the restoration results of the two methods in Figs 1–3 and Tables 1–3, we conclude that the M4-model provides a better quality of recovered images than M1-model in terms of visual quality, PSNR and RelErr. Moreover, we observe clearly that the M4-model removes noise and blur successfully and preserves more fine image details than the TV-based M1-model.

### 4.3 Comparison of M4-Model with M2-Model

In this section, we give numerical tests to compare the total generalized variation and Shearlet transform based variational model-M4 with M2-model [1] for recovering observed blurred images with multiplicative noise. For this purpose, the restoring outputs for the 256 × 256 gray level images Phantom, Cameraman and Parrot are shown. We examine the blurred and noisy images given in [1]. By means of PSNR, which is a useful tool for image quality assessment, the visual quality of the restored images is compared with M2-model quantitatively. Note that a high PSNR shows that the restoration is of good quality.

#### 4.3.1 Image Denoising

First, we show that our proposed model also provides good restoration results for multiplicative noise removal, while it is designed for the joint multiplicative denoising and deblurring. In this regard, the test images are contaminated by multiplicative noise with *L* = 10 and *L* = 6 respectively. Regarding the results obtained by the proposed model, we observe that the M4-model produces good restoration results, visually and quantitatively. Comparing with the images denoised by the M2-model given in [1], the proposed model (M4) suppresses noise and avoids staircasing effect successfully better than M2-model and preserves more fine features and details of the test images. With respect to visual quality and PSNR results shown in Figs 4–6 and Table 4, we conclude that the TGV and shearlet transform-based proposed model provides better restoration results than M2-model.

#### 4.3.2 Image Deblurring and Denoising

In this subsection, we examine the recovering of blurred images contaminated with multiplicative noise. For this purpose, two blurring functions namely, motion blur with length and angle equal to 5 and 30 and Gaussian blur with a window size and a standard deviation equal to 7 × 7 and 2, are tested respectively. Moreover, the observed images are degraded by multiplicative noise with *L* = 10 after blurring. In this case, we set 2 × 10^−5^ as the stopping criteria for our model. For our proposed model, in Figs 7–10 the degraded images and the recovered results are given, while Table 5 displays the PSNR values, the number of iterations, and CPU times. Comparing the outputs of M2-model reported in [1] with M4-model, we found that the M4-model performs quite well both quantitatively and visually. Moreover it is able to preserve more image details and features. In conclusion, our model is able to restored images successfully from multiplicative noise and blur and hence outperforms the M2-model.

### 4.4 Comparison of M4-Model with M3-Model

In this subsection, some experimental results are presented to show the performance of the M4-model. The restoration results of the M4-model are compared with M3-model [2]. We analyze the simultaneously blurred and noisy images reported in [2]. The test images are Cameraman and Parrot while the recovered images’ quality is measured by the high PSNR value.

#### 4.4.1 Image Denoising

In the first example, the performance of the M4-model is demonstrated on three types of multiplicative noise namely: Gaussian, Gamma, and Rayleigh reported in [2]. In Table 6, we have displayed the PSNR values, a number of iterations and computational CPU-time in seconds of the recovered images by M3-model and M4-model respectively. We noted that the M4-model performance in terms of PSNR outcomes is better. In Figs 11 and 12, we further show the restored images by our proposed model. It is cleared from the Table 6 and Figures (Figs 11 and 12) that the denoised outcomes of the M4-model are visually quite better than the M3-model. Recall that the recovered results of M3-model are displayed in [2].

#### 4.4.2 Image Deblurring and Denoising

In this subsection, we test the performance of our proposed model for image restoration when the observed image is corrupted by Gaussian blur, motion blur, Moffat blur and the Gamma noise simultaneously with *L* = 10. The MATLAB commands for three kinds of blurs are fspecial(‘gaussian’,[7,7],2), fspecial(‘motion’,5,30), and psfMoffat(‘[7,7],1,5) reported in [2]. In Table 7, we display the PSNR values, the number of iterations, and computational time of the recovered images by M3-model and M4-model. In Figs 13 and 14, we show the recovered images by using the M4- model, where the M3-model results are reported in [2]. It is clear from Figs 13 and 14 and Table 7, that the output results of the proposed M4-model are qualitatively and visually better than M3-model.

### 4.5 Extension to Multichannel Image Reconstruction

In this section, we extend the application of proposed model (M4) to restoring the color images corrupted with multiplicative noise and blur. We designed a set of tests in terms of different image sizes,resolutions,blurs and noise. We tested several images including Lena (dimensions = 256^2^, Bit depth = 24), parrot(dimensions = 216^2^, Bit depth = 24 and resolution = 72 dpi), eye (dimensions = 2788 × 1864, Bit depth = 24 and resolution = 300 dpi) and butterfly (dimensions = 450^2^ and Bit depth = 32), all of which have nice mixture of details, shading areas, textures and flat regions. We measured the quality of recovered images by the signal-to-noise ratio (SNR), which is measured in decibel (dB) and defined by

$$SNR=10*log_{10}\frac{∥u-\tilde{u}{∥}^{2}}{∥u-\hat{u}{∥}^{2}}$$ (27)

where *u* is the original image, $\tilde{u}$ is the mean intensity of *u* and $\hat{u}$ is the restored image. Suppose $u=\left\{u^{\left(1\right)},u^{\left(2\right)},...,u^{\left(m\right)}\right\}\in R^{mm_{1}^{2}}$ is an *m*-channel image of size $m_{1}^{2}$, where *u*^(*j*)^ represents the *j*^*th*^-channel, *j* = 1, 2, 3, …, *m* and its noisy and blurry observation $f=\left\{f^{\left(1\right)},f^{\left(2\right)},f^{\left(3\right)},...,f^{\left(m\right)}\right\}\in R^{mm_{1}^{2}}$ is given by Eq (2) in which case $H={\left\{H_{k,l}\right\}}_{k,l=1}^{m}\in R^{mm_{1}^{2}\times mm_{1}^{2}}$ is a cross-channel blurring operator. For instance, in the standard RGB (red,green and blue) color system, the image $u=\left\{u^{\left(r\right)},u^{\left(g\right)},u^{\left(b\right)}\right\}\in R^{3m_{1}^{2}}$. We restore *u* via the following problem

$$\begin{array}{c} \underset{u,p}{min}E_{H}\left(u\right)=\sum_{j=0}^{m}\left[\langle log\left(Hu^{\left(j\right)}\right),1\rangle +\langle \frac{f^{\left(j\right)}}{Hu^{\left(j\right)}},1\rangle \right]+\alpha \sum_{j=0}^{m}{∥{\left(\sqrt{\frac{Hu^{\left(j\right)}}{f^{\left(j\right)}}}-1\right)}^{2}∥}_{2}^{2}+\lambda \sum_{j=0}^{N}\sum_{j=0}^{m}{\left|{\text{SH}}_{j}\left(u^{\left(j\right)}\right)\right|}_{1}\\ +\gamma_{1}\sum_{j=0}^{m}∥\nabla u^{\left(j\right)}-p^{\left(j\right)}{∥}_{1}+\gamma_{0}\sum_{j=0}^{m}{∥\bar{\varepsilon }\left(p^{\left(j\right)}\right)∥}_{1} \end{array}$$ (28)

where *m* represents number of layers of a vectorial image. The derivation of algorithm is similar to that of Algorithm-1. To demonstrate the performance of the proposed model (M4), we ran four tests with within and cross-channel blurs. Let the cross-channel kernel *K* be of the form

$$K=\left(\begin{array}{ccc} K_{11} & K_{12} & K_{13}\\ K_{21} & K_{22} & K_{23}\\ K_{31} & K_{32} & K_{33} \end{array}\right)$$

where *K*_*ij*_, *i* = *j* are within channel kernels and *K*_*ij*_, *i* ≠ *j* are cross-channel kernels. For simplicity, let M(length,theta) be the motion blur with a motion length and an angle theta in the anticlockwise direction. Likewise, G(hsize,sigma) be the Gaussian blur with a square support size ‘hsize’ and standard deviation ‘sigma’, and the average blur A(hsize) with a square support size ‘hsize’. In our tests, we generated cross-channel blurring kernels in the same way as in [57] which is given below

- Generate 9-kernels: [*M*(11, 45), *M*(21, 90), *M*(41, 135), *G*(7, 5), *G*(9, 5), *G*(11, 5), *A*(13), *A*(15), *A*(17)],
- Randomly assign the above 9-kernels to *K*_11_, *K*_12_, *K*_13_, *K*_21_, *K*_22_, *K*_23_, *K*_31_, *K*_32_, *K*_33_,
- Multiply each *K*_*ij*_ with *ω*_*ij*_, where $W\in R^{3^{2}}$ and *ω*_*ij*_ is the (*i*, *j*)^*th*^ element of *W*.

Note in the tests, we used within-channel blurring that is setting *W* to be the unit matrix of order-3 and the cross-channel kernels with

$$W=\left(\begin{array}{ccc} 0.71 & 0.15 & 0.15\\ 0.12 & 0.80 & 0.20\\ 0.30 & 0.30 & 0.60 \end{array}\right)$$

**Fig 15:**
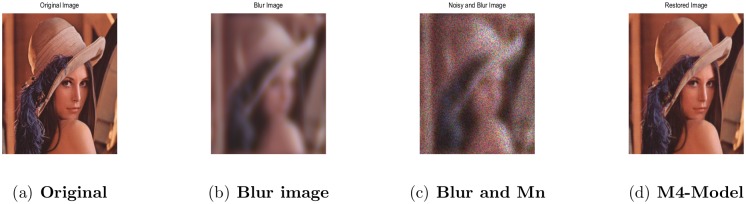
RGB Lena image 256^2^ result from the proposed model (M4). (a) original Lena image; (b) blur image blurred by the within-channel kernel; (c) the observed image degraded by blurring operator and the multiplicative noise with *σ*^2^ = 0.1; (d) the restored image by our proposed model (M4).

All parameters were set to be identical to those described in Section-4.1. In the first experiment, we used within-channel blurring i.e, setting *W* to be the identity matrix of order-3 to generate a blurry image and then corrupted its pixels by multiplicative noise (also known as speckle) with *σ*^2^ = 0.1. The restored image in Fig 15 and the SNR result given in Table 8, clearly demonstrate the good restoration performance of the proposed model (M4).

**Table 8:** Restoration results of the proposed model (M4) for multiplicative deblurring, evaluated using SNR, number of iterations and CPU-time in seconds.

Image/Size	Blur Operator	Noise	M4-Model		
			SNR	Iter.	Time
Lena/256^2^	within-channel kernel	*σ*^2^ = 0.1	14.39	100	45.94
parrot/216^2^	cross-channel kernel	*σ*^2^ = 0.2	13.20	154	69.05
eye/2788 × 1864	cross-channel kernel	*σ*^2^ = 0.1	21.97	385	134.66
butterfly/450^2^	cross-channel kernel	*σ*^2^ = 0.2	12.28	168	87.90

**Fig 16:**
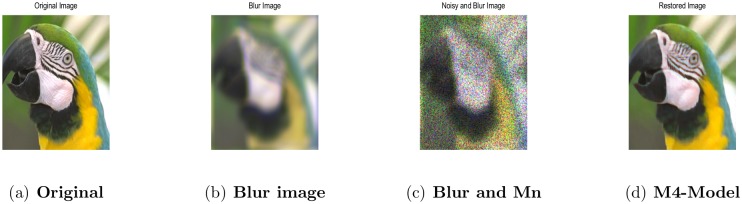
Performance of M4-model on color parrot image 216^2^. (a) original image; (b) blur image contaminated by the cross-channel kernel; (c) the observed image degraded by blur and the multiplicative noise with *σ*^2^ = 0.2; (d) the restored image by our proposed model (M4).

In addition to the grayscale image parrot, we also tested an RGB image parrot (216^2^). we first generated a blurry image by the cross-channel kernel and then is contaminated by multiplicative noise with *σ*^2^ = 0.2. From Table 8 and Fig 16, it is obvious that the proposed model (M4) achieves the desirable outcome in terms of SNR and qualitative results, which validates the effectiveness of the proposed model for color images.

**Fig 17:**
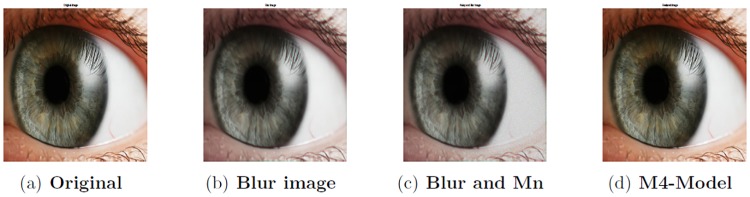
Performance of M4-model on color eye image 2788 × 1864. (a) original image; (b) blur image corrupted by the cross-channel kernel; (c) the observed image degraded by blurring kernel and the multiplicative noise with *σ*^2^ = 0.1; (d) the restored image by our proposed model (M4).

**Fig 18:**
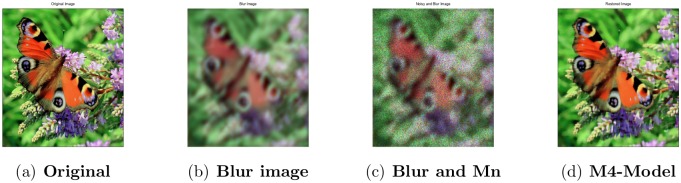
Performance of M4-model on color butterfly image 450^2^. (a) original image; (b)blur image corrupted by the cross-channel kernel; (c) the observed image degraded by blur and the multiplicative noise with *σ*^2^ = 0.2; (d) the restored image by our proposed model (M4).

In the third and fourth experiments, an eye and butterfly color images are corrupted by cross-channel kernel and multiplicative noise with *σ*^2^ = 0.1, 0.2 respectively. The denoising results shown in Figs 17 and 18 and SNR values in Table 8, clearly show the desirable results of the proposed model (M4). The object edges are well preserved and staircase minimizes, leading to a more faithful denoising for real images.

## 5 Conclusion

This paper describes a new variational model-M4 to removing blur and multiplicative noise simultaneously. The proposed model combines a total generalized variational filter with shearlet transform. We developed an efficient alternating direction method of multipliers (ADMM) for the solution of optimization problem arisen from the minimization of proposed energy functional. We analyze the blurred images and noisy images reported in [1, 2, 38], consisting of smooth regions and edges. From the numerical results, we found that the proposed model-M4 is able to preserve sharp edges like the other models while at the same time reducing the blocky effects in smooth regions better than the recent variational models. In a word, the combined method utilizes benefits of both the total generalized variation penalty and shearlet transform in image restoration task. Extensive experiments on real vectorial images validate the very good performance of the proposed model. The idea in the work can be extended to many other mathematical problems in image processing. Developing fast algorithms for solving differential equations arisen from model minimization and to adopt the automated spatially dependent regularization parameter selection framework for image restoration, might be considered in future work.
